# Translational strategies to uncover the etiology of congenital anomalies of the kidney and urinary tract

**DOI:** 10.1007/s00467-024-06479-2

**Published:** 2024-10-07

**Authors:** Lisanne M. Vendrig, Mayke A. C. ten Hoor, Benthe H. König, Iris Lekkerkerker, Kirsten Y. Renkema, Michiel F. Schreuder, Loes F. M. van der Zanden, Albertien M. van Eerde, Sander Groen in ’t Woud, Jaap Mulder, Rik Westland, on behalf of the ArtDECO consortium, L. S. Klomp, L. M. Mak-Nienhuis, R. F. J. Marsman, L. A. Groen, D. Bourjouane, M. W. T. Tanck, J. W. Groothoff, E. Levtchenko, A. S. Brooks, J. R. Scheepe, V. V. A. M. Knoers, P. Deelen, L. H. Franke, R. W. G. van Rooij, H. S. Spijker, C. W. van den Berg, R. Bijkerk, P. Hohenstein, A. J. Rabelink, W. F. J. Feitz, N. Roeleveld, I. A. L. M. van Rooij, G. Schijven, S. Teuben, E. van de Geer-de Jong, J. A. Schulp, A. J. Klijn, K. D. Lichtenbelt, M. N. Bekker, G. van Haaften, M. R. Lilien

**Affiliations:** 1 Department of Pediatric Nephrology, Amsterdam UMC-Emma Children’s Hospital, University of Amsterdam, Meibergdreef 9, 1105 AZ Amsterdam, The Netherlands; 2 Division of Nephrology, Department of Pediatrics, Willem-Alexander Children’s Hospital, Leiden University Medical Center, Leiden, The Netherlands; 3 Department of Human Genetics, Leiden University Medical Center, Leiden, The Netherlands; 4 IQ Health Science Department, Radboud University Medical Center, Nijmegen, The Netherlands; 5 Department of Genetics, University Medical Center Utrecht, Utrecht, The Netherlands; 6 Department of Pediatric Nephrology, Amalia Children’s Hospital, Radboud University Medical Center, Nijmegen, The Netherlands; 7 Department of Human Genetics, Radboud University Medical Center, Nijmegen, The Netherlands; 8 Division of Nephrology, Department of Pediatrics, Sophia Children’s Hospital, Erasmus Medical Center, Rotterdam, The Netherlands

**Keywords:** CAKUT, Genetics, Environmental hazard exposure, Model systems

## Abstract

While up to 50% of children requiring kidney replacement therapy have congenital anomalies of the kidney and urinary tract (CAKUT), they represent only a fraction of the total patient population with CAKUT. The extreme variability in clinical outcome underlines the fundamental need to devise personalized clinical management strategies for individuals with CAKUT. Better understanding of the pathophysiology of abnormal kidney and urinary tract development provides a framework for precise diagnoses and prognostication of patients, the identification of biomarkers and disease modifiers, and, thus, the development of personalized strategies for treatment. In this review, we provide a state-of-the-art overview of the currently known genetic causes, including rare variants in kidney and urinary tract development genes, genomic disorders, and common variants that have been attributed to CAKUT. Furthermore, we discuss the impact of environmental factors and their interactions with developmental genes in kidney and urinary tract malformations. Finally, we present multi-angle translational modalities to validate candidate genes and environmental factors and shed light on future strategies to better understand the molecular underpinnings of CAKUT.

**Graphical abstract:**

**Figure:**
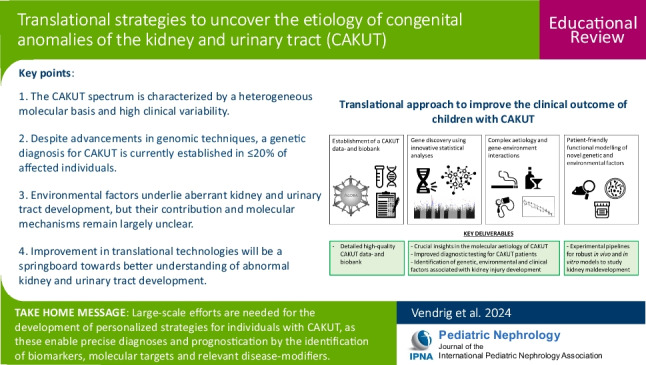
A higher resolution version of the Graphical abstract is available as Supplementary information

**Supplementary Information:**

The online version contains supplementary material available at 10.1007/s00467-024-06479-2.

## Introduction

Congenital anomalies of the kidney and urinary tract (CAKUT) represent a spectrum of human developmental defects caused by aberrations during embryonic kidney and urinary tract formation [1–3]. The definitive development of the kidney and urinary tract in humans starts at Carnegie stage 14 (i.e., gestational week 5) [4, 5]. During nephrogenesis, the ureteric bud interacts with the metanephric mesenchyme in a spatiotemporal context to form the definitive kidneys. Any disruption within this process may result in kidney anomalies (KA; e.g., kidney agenesis, multi-cystic dysplastic kidney (MCDK), kidney dysplasia, kidney hypoplasia, ectopic kidney tissue, or duplex kidney), obstructive nephropathy (e.g., ureteropelvic junction obstruction (UPJO), ureterovesical junction obstruction, unspecified congenital hydronephrosis), and/or vesicoureteral reflux (VUR). Lower urinary tract obstruction (e.g., posterior urethral valves (PUV)) is hypothesized to originate from aberrant insertion of the mesonephric duct into the urogenital sinus [6].

Although CAKUT underlies up to 50% of pediatric kidney failure [7], clinical outcomes of CAKUT vary greatly with the vast majority of patients facing a relatively mild prognosis [8, 9]. This underlines the need for precision medicine approaches to achieve accurate diagnoses and individual prognostication for patients. Studying the molecular etiology of CAKUT may potentially improve the clinical outcome of patients in a similar manner as for other kidney diseases [10, 11]. In this review, we discuss translational strategies to elucidate the genetic and environmental underpinnings of CAKUT and highlight future perspectives to devise personalized clinical management.

## Rare variants in genes of kidney and urinary tract development and copy number variants make up the current genetic architecture of CAKUT

Given the early observations of familial segregation in 15–20% of CAKUT cases [1, 2, 8], a genetic basis for abnormal kidney and urinary tract development has long been proposed. Other evidence for monogenic forms of CAKUT includes the existence of corresponding monogenic mouse models [12] and the presence of monogenic syndromes that include CAKUT [13]. CAKUT could be caused by rare single nucleotide variants (SNVs) or copy number variants (CNVs, e.g., microdeletions or microduplications) with or without known genes that play a role during nephrogenesis and urinary tract development (Supplementary Table 1&2; Supplementary Data) [2]. These disease-causing SNVs and CNVs have large effects and mainly follow a Mendelian inheritance pattern [14]. The eligibility criteria for clinical genetic testing in CAKUT has been elegantly described elsewhere [15] and include factors that increase the probability for the identification of monogenic CAKUT.

### Chronological overview of rare genetic and genomic variation in CAKUT

Early genetic studies focused on syndromic forms of CAKUT [1], showing the association of CAKUT with larger chromosomal anomalies, such as trisomy 21 and Turner syndrome (5% and 17–42% of affected individuals have CAKUT, respectively) [16, 17]. Additional studies identified genomic variation in key genes involved in kidney and urinary tract development. These included SNVs in genes encoding transcription factors and signaling molecules (e.g., *PAX2* [18], *HNF1B* [19], *SIX1* [20], *EYA1* [21], *RET* [22], and *FGF20* [23]).

The significance of structural variation in the etiology of CAKUT has been emphasized by studies that found a higher burden of exonic CNVs and an enrichment of known genomic disorders (caused by larger CNVs) in CAKUT cases in comparison to healthy controls (Supplementary Table 2) [14]. Specifically, large and rare deletions were proposed as the underlying mechanism behind genomic disorders associated with KA, including renal cysts and diabetes syndrome and Alagille syndrome [24, 25]. The ability to pinpoint causal genes within identified CNV regions has subsequently resulted in valuable new insights in the etiology of CAKUT, for example, by the identification of *TBX6* and *MAZ* as driver genes for CAKUT in individuals with the chromosome (Chr.)16p11.2 microdeletion syndrome [14, 26, 27] as well as *HNF1B* and *CRKL* for Chr.17q12 and Chr.22q11.2, respectively [24, 28, 29].

**Fig. 1:**
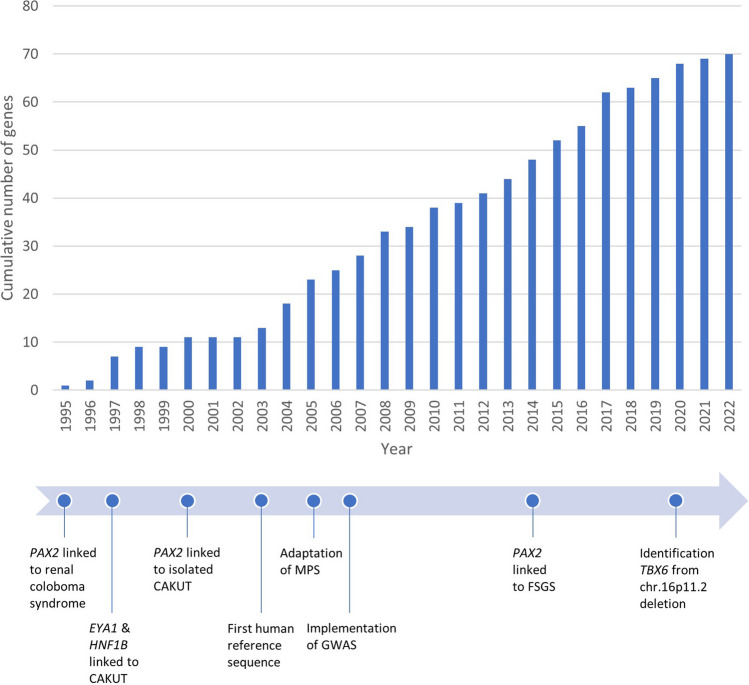
Selected genetic milestones and cumulative numbers of genes discovered for CAKUT over time. This figure is a CAKUT-specific update from [31]. The genes cumulatively counted are based on the CAKUT panel from Genomics England PanelApp, Table S1 from [13], and the diagnostic experience in our local university medical centers. Publication years are based on discovery articles and Online Mendelian Inheritance in Man (OMIM). *CAKUT*, congenital anomalies of the kidney and urinary tract; *FSGS*, focal segmental glomerulosclerosis; *MPS*, massive parallel sequencing; *GWAS*, genome-wide association study

The adoption of massive parallel sequencing (MPS) technologies, including whole-exome sequencing (WES) and whole-genome sequencing (WGS), has fueled the current identification of more than 60 genes linked to both syndromic and isolated CAKUT (Supplementary Table 1, Fig. 1) [30, 31]. Nevertheless, a molecular diagnosis is detected in maximum 20% of patients with CAKUT, depending on cohort characteristics [2, 32]. In addition to observed incomplete or non-penetrance of disease-causing SNVs [33], variants in the same genes may underlie different CAKUT phenotypes (i.e., variable expressivity), even within families [34]. Both incomplete penetrance and variable expressivity suggest pivotal roles for epigenetic mechanisms and co-regulation of multiple genes as well as environmental factors in the molecular etiology of CAKUT [1, 35, 36] and form one of the main challenges for the detection of pathophysiological mechanisms in CAKUT [37]. By sequencing more patients in larger cohorts with syndromic and isolated CAKUT as well as the establishment of large MPS public datasets such as gnomAD [38], the clinical variability of genetic forms of CAKUT and their genotype–phenotype correlations are likely to become better defined.

### WGS and CAKUT

At the moment, WGS is not routinely applied in genetic testing for CAKUT, but its implementation has been initiated. WGS allows for the identification of a wide range of disease-causing variants, including coding and non-coding SNVs and CNVs, structural rearrangements, and repeated sequences [39–41]. Studies have shown that WGS has a diagnostic yield of about 25% for patients with rare diseases, with more than 10% of these diagnoses attributed to genomic SNVs in regions not detected by methods other than WGS [40, 42]. A large pilot study within the Genomics England Project focusing on patients with undiagnosed rare diseases offered a diagnostic yield of around 7% for CAKUT cases [40], which was comparable to several WES-based studies [43–45]. The molecular diagnoses in this study were mainly detected in regions that would have been covered by WES. Notably, the highest yield was observed in analyses involving patient–parent trios, outperforming those involving only the proband or more family members [40]. In a study in the same cohort in boys with PUV, specific enrichment of inversions that affect chromatin looping was found with WGS [46]. This further underlines the potential of WGS to improve our understanding of the molecular mechanisms of CAKUT, especially when trios are selected. However, interpreting WGS data beyond the coding regions remains challenging and requires, among others, the integration with additional “omics” data, such as epigenomics, transcriptomics, and proteomics [8, 41]. For detailed mapping and identification of relevant gene regulatory regions, data from the Encyclopedia of DNA Elements (ENCODE) Project Consortium could be used [47]. Additionally, the vast amount of data produced by WGS requires significant computational power and storage capacity, which include a logistical challenge and, potentially, a negative ecological impact when applied on a global scale [48].

## The role of common variants in the etiology of CAKUT remains largely elusive

As highlighted in the previous section, many SNVs and CNVs have been linked to either syndromic or isolated forms of CAKUT [2]. Because these genetic disorders are under strong selective pressure, they are predominantly found in rare CAKUT phenotypes such as KA or UPJO. On the contrary, the prevalence of more common CAKUT phenotypes, such as low-grade VUR, duplex kidney, or ectopic kidney tissue, might be better explained by one or more common variants with a smaller effect size [49].

To identify common variants associated with CAKUT, genome-wide association studies (GWAS) may be performed. In GWAS, the association between single nucleotide polymorphisms (SNPs) and the phenotype of interest is assessed with genome-wide genotyping using chromosomal microarrays (CMA) or WGS. WGS has a higher genomic resolution, does not require genomic imputation, and potentially enables the discovery of rare variants compared to CMA.

Several studies have reported on the role of common variants across different CAKUT phenotypes (Table 1), although associations were not robust. The lower yield for GWAS in CAKUT, compared to other kidney diseases [50, 51], is likely due to the relatively high phenotypic heterogeneity within study cohorts and low sample sizes. Taking into account the variable expressivity of CAKUT [34], one could suggest a common genetic basis and advocate for a GWAS that combines all CAKUT phenotypes [52]. On the other hand, this phenotypic heterogeneity could also complicate finding genetic associations, as they may be well-explained by rare variants with small-to-moderate effect sizes for which the implication of causality is more challenging. To visualize this complexity of assigning genetic causality in CAKUT, Martino et al. calculated incredible estimates of 93,384 cases and 9,244,968 controls needed to identify genome-wide significant associations in a theoretical but feasible scenario that includes a disease prevalence of 1.0% (e.g., VUR or duplex kidney), effect size of OR = 1.5, and a minor allele frequency of 0.1% [37]. Although average sample size in GWAS has generally increased rapidly over the past few years, CAKUT is lagging behind and the establishment of larger biobanks is greatly needed. Another limiting factor in the generalizability of current GWAS is the predominance of cases and controls of European ancestries, which has introduced bias due to differences in allelic variation across populations (i.e., much higher in sub-Saharan African populations compared to Caucasian populations) [53]. Both limitations of GWAS in CAKUT indicate the need for large and global collaborations.

**Table 1:** Overview of GWAS in CAKUT

Phenotype	Ref	Year	Study population	Cases (*n*)	Controls (*n*)	Technology	Summary of findings
Congenital solitary functioning kidney	[54]	2022	Netherlands	452	669 + 5363	SNP array	Chr.7, rs140804918; OR 3.1. Candidate gene: *HGF*, expressed in kidney tissue during embryonic kidney development [55, 56] Chr.18, rs184382636; OR 12.9. No candidate gene 30 additional suggestive loci
Vesico-ureteral reflux	[57]	2021	European (North-western European, Polish, Italian, and South-eastern European)	Total: 1395 Females: 937 Males: 458	Total: 5366 Females: 3129 Males: 2235	SNP array	Total population: Chr.2p15, rs13013890; OR 3.65, 95% CI 2.395.56. Candidate gene: *WDPCP*, involved in Bardet-Biedl syndrome, which can encompass kidney anomalies (OMIM #615992) In Females: Chr.6p12.1, rs1154855; OR 1.41, 95% CI 1.26–1.59. Candidate gene: *BMP5*, involved in early nephrogenesis [58] In Males: Chr.6q14.1, rs10806089; OR 2.75, 95% CI 1.96–3.84. Candidate gene: *HTR1B*, expressed in cells of bladder mesenchyme and urethra [57] 5 additional suggestive loci
	[59]	2010	UK	348	2938	SNP array	Suggestive involvement of three loci: Chr.10, rs1159217 Chr.11, rs17306391 Chr.18, rs12604993
	[60]	2017	Ireland, UK, and Slovenia	1147	3789	SNP array	No association results
	[61]	2012	Netherlands	207 (with or without duplex kidney)	554	SNP array	No significant associations; suggested involvement of *GREM1*, *EYA1*, *ROBO2*, and *UPK3A*
Posterior urethral valves	[62]	2022	European (Netherlands, German and Polish)	756 cases	4823	SNP array	No significant associations; 33 suggestive variants
	[46]	2022	Mixed ancestry	132	23,727	WGS	Chr.12q24.12, rs10774740; OR 0.4, 95% CI 0.31–0.52. Candidate gene: *TBX5* Chr.6p21.1; rs144171242; OR 7.2, 95% CI 4.08–12.70. Candidate gene: *PTK7* Proteins of both of these genes have been detected in the developing urinary tract [46]

*Chr.* chromosome, *CI* confidence interval, *OR* odds ratio, *SNP* single nucleotide polymorphism, *WGS* whole genome sequencing

## Environmental factors in maternal health and lifestyle during pregnancy are associated with CAKUT

Besides genetic factors, the role of environmental factors in the etiology of CAKUT has been the subject of several, mostly epidemiological, studies, all of which are characterized by relatively small samples sizes, differences in the included CAKUT phenotypes, and inconsistent outcomes [63, 64] (Table 2). Because these studies report merely statistical associations, the molecular mechanisms of how exposure to the proposed environmental factors leads to CAKUT remain largely speculative.

**Table 2:** Overview of environmental risk factors in the etiology of CAKUT

Maternal health factors	Strength of association
Obesity	+ +
Increased age	+ / −
Ethnicity	?
Gravidity	?
Diabetes	+ +
Gestational diabetes	+ / −
Subfertility	+
Infections	+
Maternal exposures during pregnancy	
Smoking	+ / −
Alcohol	+ / −
Folic acid use	+ / −
Medication use (ACEi, retinol)	?

+  + increased risk, multiple studies; + increased risk, few studies; + / − conflicting evidence; ? unknown. *ACEi* angiotensin-converting enzyme inhibitors

### Maternal health factors (during pregnancy)

The most studied potential risk factor for CAKUT is maternal obesity. In 2021, Jadresić et al*.* performed a meta-analysis which showed an increased risk of having a child with CAKUT when mothers were obese (odds ratio (OR) 1.14, 95% confidence interval (CI) 1.02–1.27) [65]. Other studies confirmed this association [64, 66, 67], which is hypothesized to be caused by higher blood glucose levels that may interfere with ureteric budding [68], as well as with increased inflammation and changes in steroid hormone levels [69, 70].

Maternal age at pregnancy has also been associated with a risk of CAKUT in offspring, although the few conducted studies show conflicting results. Some studies have found a higher risk for older mothers [71], whereas others reported an increased risk for younger mothers [72, 73]. As such, definitive conclusions on the role of maternal age in CAKUT etiology cannot be drawn yet. In addition, maternal ethnicity and gravidity remain to be studied [74].

Both pre-existing diabetes mellitus (DM) and gestational diabetes mellitus (GDM) have been analyzed as risk factors for having a child with CAKUT [67, 75–78]. Several studies report a consistent significant association between mothers with DM and KA in their offspring (OR range 2.4 (95% CI 1.3–5.6)–10.5 (95% CI 4.0–27.2)) [67, 75, 79, 80]. Additional studies investigating DM as a risk factor for all CAKUT phenotypes found weaker, but still statistically significant associations [77, 78, 81, 82], whereas others did not [63, 71]. The same inconsistency was found for the role of GDM as a risk factor for CAKUT: while some studies showed an increased risk for CAKUT in children from mothers with GDM [71, 82], other studies did not confirm this association [67, 77, 83]. Potentially, the timing of onset of GDM during pregnancy may explain this inconsistency, which could affect different stages of nephrogenesis and therefore lead to different CAKUT phenotypes (e.g., early-onset GDM may have large effects on kidney tissue formation, whereas later-onset GDM may affect total nephron number endowment only).

The few studies investigating the association between subfertility and CAKUT found varying results [64, 84, 85], which is partly explained by the use of different definitions of subfertility. Standardized and systematic research is needed in order to determine whether subfertility is an attributing or confounding factor in the etiology of CAKUT. This also holds true for infections during pregnancy [86, 87].

### Maternal exposures during pregnancy

Surprisingly, the association between smoking and alcohol consumption during pregnancy and CAKUT is much less established than for other human developmental defects [63, 72, 81, 88–92]. The well-known protective effect of folic acid use during pregnancy for neural tube defects [93] is also reported for CAKUT [64, 94–96]. However, not all studies focusing on CAKUT observed this association [64, 84, 85], implicating specificity during a limited time period in kidney and urinary tract development. Although the effect of several drugs (e.g., angiotensin-converting enzyme inhibitors, retinol, antiepileptic drugs) on kidney development has been extensively reviewed [97], the mechanisms explaining how maternal medication during nephrogenesis leads to CAKUT in the offspring have not yet been systematically studied.

### Gene–environment interactions

Gene–environment interactions have been established for other human developmental defects such as cleft lip and hypospadias, that, similar to CAKUT, are characterized by a complex genetic architecture [98, 99]. A study by members from our group has found an interaction between obesity and the genetic variant rs3098698, located in an intron of *ARSB* on Chr.5, which increased the risk of KA [100]. However, no other studies have currently investigated such interactions for CAKUT, probably due to the incomplete simultaneous recording of genetic and environmental factors by study protocols. As the methodologies to identify relevant gene–environment interactions in health and disease are improving [101], we recommend future studies to also collect exposome data (i.e., the impact of environmental exposures on one individual in a lifetime) in addition to genetic data and environmental exposures during nephrogenesis. This would create detailed CAKUT cohorts that allow for the gene–environment studies necessary to fill this knowledge gap in the molecular mechanisms of CAKUT.

## Functional validation methods uncover the molecular mechanisms of CAKUT from multiple angles

The identification of candidate molecular factors in disease etiology includes the challenge of drawing definitive conclusions about true causality. However, this is a prerequisite for the implementation of such findings into clinical management, e.g., by the inclusion in diagnostic test panels or risk assessment scoring modalities. While the pathogenicity of genetic variants could be further substantiated by replication of findings in other datasets, information on the type of finding (de novo, allele frequency, segregation), and in silico predictions [102], in vitro and in vivo models remain the cornerstones to establish causality. In-depth functional analysis also harbors the potential to identify new molecular pathways and key molecules that orchestrate kidney and urinary tract development. In this manner, discovery of non-modifiable genetic causes of CAKUT may identify potentially targetable up- or downstream mediators [103].

### Model systems

The choice of model systems for functional validation primarily depends on the clinical scenario or phenotype to be investigated. The different in vitro and in vivo models each provide their own set of read-outs and degree of similarity to the organ system, which will be discussed in more detail. To mimic the effects of environmental factors, model systems can be exposed to these factors via maternal introduction for mammalian embryos or via culture medium for larval (fish, amphibian) and in vitro models. For genetic variants, model system choice also depends on the actual discoveries at hand. Ideally, the gene of interest is present within the model system and the actual variant conserved. A solution to the lack of an orthologous gene can be the generation of a knock-in model, as has been done for instance for the entire (non-CAKUT) *APOL1* in mice [104], while individual codons have also been humanized in mice to investigate specific gene functions [105]. Currently available (permanent) gene-editing techniques (e.g., CRIPR/Cas9) greatly facilitate the generation of such tailor-made models. On the other hand, one needs to be aware that the presence of paralogous, duplicate genes may limit the experimental applicability of an otherwise suitable model system. Since normal development is highly dependent on specific spatial and temporal gene expression patterns, thorough CAKUT modeling often relies on the use of conditional gene expression models using Cre-recombinase mouse lines that enable tissue- and/or developmental stage-specific experimental interventions. Other techniques allow for (more rapid) assessment of temporary suppression (e.g., morpholinos) or induction of gene expression (e.g., plasmids or viral vectors), which are also useful for screening candidate genes [106–108].

### In vitro and in vivo techniques

Culture of cells derived from the kidney or specific structures within the urinary tract can be applied to study gene expression, gene function, and molecular pathways at the single-cell level. Benefits include the ease of genetic manipulation and exposure to environmental factors and the availability of immortalized and primary cell lines, which include both kidney epithelial cells and urothelial cells [107, 109]. An inherent drawback of culturing individual cell types is the lack of recapitulation of interaction between different cell types, which is an essential aspect of (ab)normal kidney development. Kidney organoids, mini-3D structures that contain as many as 20 different kidney cell types, may provide a suitable alternative for this problem. They are derived from either human embryonic stem cells or induced-pluripotent stem cells (hiPSC) and resemble the cellular composition and expression profile of early second-trimester human fetal kidney, providing a valuable, complementary methodology to study early kidney development [110, 111]. For modeling genetic variants, stem cells can be genetically modified or, in the case of hiPSCs, generated from a multitude of actual patients’ somatic cell types (e.g., skin fibroblasts, blood or urine cells). Using patient-derived kidney organoids and isogenic controls could improve the accuracy of modeling by providing the appropriate (epi)genetic context of the studied disease. In addition, complex techniques to profile gene expression (e.g., single-cell RNA sequencing) or genome-wide protein-DNA interactions (e.g., chromatin immunoprecipitation sequencing) may reveal novel transcriptional mechanisms involved in disease [112]. However, the current applications of organoids for CAKUT are limited to modeling kidney hypoplasia or kidney dysplasia phenotypes and, therefore, do not include other kidney malformations or urinary tract defects such as obstruction, vesicoureteral reflux, and bladder abnormalities due to their respective challenges regarding further maturation, vascularization, and anatomical integrity [113].

Animals remain indispensable in CAKUT research to mimic the complete multi-organ urinary system and the complexity of the human kidney. The mouse (*Mus musculus*) is the predominant in vivo model system in kidney research because of its similarity to human kidney development and the extensive research tools available. Mouse-based research has led to the validation and elucidation of multiple genes involved in CAKUT (e.g., *PAX2* and *HNF1B*), as is comprehensively reviewed elsewhere [114]. The functional validation of a(n) (epi)genetic or environmental factor can be performed via assessment of the phenotype at one or serial developmental time-points. Whereas the initial evaluation often comprises more traditional techniques like immunohistochemistry and gene/protein expression profiling, in-depth mechanistic analysis involves genetic tools like lineage tracing and fluorescent reporters to track the progeny of specific cells and visualize spatiotemporal gene expression patterns [115]. The combination of these genetic tools with ex vivo culture of embryonic kidneys facilitates the dynamic study of multiple embryonic stages from a single embryo [116].

The zebrafish (*Danio rerio*) has proven to be a robust vertebrate model for studying kidney developmental defects. Its large number of offspring (*n* ~ 50–200) and the conservation of 82% of human disease-related genes allow for less expensive and higher-throughput screenings compared to mice. Although zebrafish do not develop a metanephric kidney, the larval pronephros is useful for kidney research as a simplified version of the human nephron [108, 117]. The larvae develop ex utero and are translucent, enabling rapid phenotype analysis and live imaging of fluorescent reporters. The effect of environmental factors on the kidney has been successfully studied in zebrafish, which may also allow for the investigation of gene–environmental interactions in CAKUT [118, 119]. Research on genetic variants has demonstrated that zebrafish are suitable to test disease relevance and direction of effect. For example, morpholino-induced loss of function in zebrafish embryos has been used to screen several candidate genes for Chr.22q11.2 microdeletion syndrome [29]. Furthermore, modeling CAKUT in zebrafish generates the possibility of testing causality of individual SNVs [120]. However, the duplicated genome in zebrafish makes them solely suitable for candidate genes without paralogous genes, as these could compensate for the effects of gene manipulation.

## Future translational approaches to uncover the etiology of CAKUT

Despite the increasing insights into (ab)normal kidney and urinary tract development, the molecular etiology is still not identified for the vast majority of CAKUT patients.

With the lowering costs of WGS, this technique will likely replace WES as the standard method of choice to identify rare and common disease-causing variants in CAKUT. Several challenges of WGS data analysis and interpretation (especially in singletons) and storage still need to be solved. Moreover, the bioinformatic analysis of WGS data is highly intensive computationally, which increases its costs. However, it is expected that artificial intelligence technologies may resolve many of these problems [121]. Beyond WGS, even newer and more comprehensive methods like long-read sequencing and Hi-C await [122–124]. Furthermore, the recent introduction of spatially resolved transcriptional technologies may uncover the transcriptome and proteome during different stages of (disturbed) nephrogenesis at a single-cell resolution. Not only would this uncover the relevant pathomechanisms of CAKUT caused by genetic conditions in a spatiotemporal manner but, potentially, also provide better insights in the altered molecular mechanisms initiated by exposure to environmental or epigenetic factors during nephrogenesis. Major hurdles to the use of spatially resolved transcriptomics in CAKUT are the fact that these technologies require extremely rare human tissues from the kidney and urinary tract, preferably during gestation. Of note, this is also the predominant reason why research efforts that study the role of epigenetic factors in the pathogenesis of CAKUT have been very scarce, especially since a kidney biopsy is a relative contra-indication in the clinical management of CAKUT. The lack of kidney tissue in CAKUT may be circumvented by using single-cell RNA sequencing of urine-derived kidney cells from newborns with CAKUT to partly overcome these hurdles. Such a patient-friendly and non-invasive “liquid kidney biopsy” is based on the observation that kidney cells, e.g., podocytes and proximal tubular cells, are more abundantly excreted in the urine of individuals affected by kidney diseases (such as cystinosis, podocytopathies, acute kidney injury) than of healthy controls [125]. However, it is still unknown if relevant pathomechanisms remain detectable in kidney cells after nephrogenesis has been completed.

**Fig. 2:**
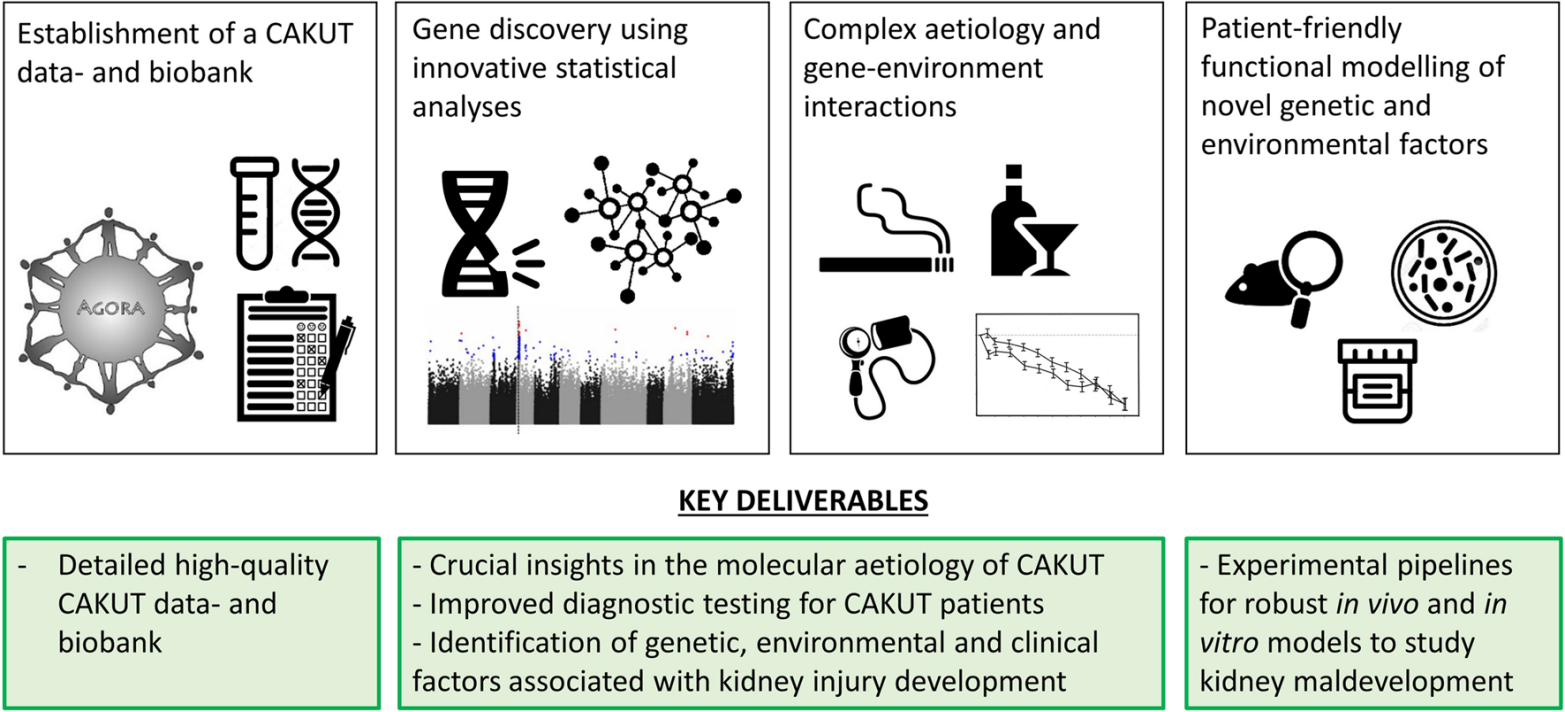
Framework of the ArtDECO research consortium. ArtDECO aims to integrate highly detailed phenotypic and clinical information of affected individuals with genetic data (generated by whole genome sequencing and chromosomal microarrays) with environmental exposure data to uncover the molecular etiology of CAKUT and improve the clinical management of patients. Candidate factors are subsequently validated by using tailor-made model systems

Although the general prognosis of CAKUT has improved tremendously, the knowledge gap in the etiology impedes further enhancement of clinical outcomes for affected individuals, as this requires personalized approaches. We have therefore founded the Aetiology of renal and urinary tract anomalies defines Diagnostic Efficacy and Clinical Outcome (ArtDECO) consortium, a large translational research framework within the Netherlands that includes a data- and biobank with clinical, genetic, and environmental exposure data from *n* ~ 3750 individuals with different CAKUT phenotypes and their family members (Fig. 2). By using WGS, we aim to provide broader insights into the genetic architecture of CAKUT. The collected clinical data include laboratory measurements, anthropometric measurements, (prenatal and postnatal) imaging data, and demographical and perigestational questionnaire data, such as prevalence of infection, smoking, and alcohol consumption. The combined use of these data will allow for studies into environmental factors and their interaction with genomic variation. Identified novel genetic and environmental factors will be functionally studied using in vivo and in vitro model systems to understand their roles in abnormal kidney and urinary tract development. For this purpose, we aim to use patient-friendly methods by creating kidney organoids from iPSCs from the urine of patients. With our strategy to integrate detailed phenotypic information of patients with genetic and environmental exposure data, we aim to enhance clinical diagnostic techniques and prognostic modeling for individual patients. Paradoxically, cohorts of probably 10,000s of affected individuals are required to ultimately develop a personalized strategy that improves the prognostication and clinical outcome of individuals with CAKUT. We realize that this goal requires collaboration on an international scale. As such, we aim to contribute to the establishment of highly needed diverse CAKUT cohorts for which multi-angle data are available for the uncovering of the complex molecular underpinnings of CAKUT.

## Conclusions

In summary, the multi-factorial molecular pathogenesis of CAKUT encompasses different human genetic paradigms, as well as intrinsic and extrinsic environmental hazard exposures. The role of epigenetic factors in disturbed nephrogenesis remains a topic of future studies. Although our understanding of the etiology of CAKUT has grown due to the increasing resolution of translational techniques, progress has been slower than for many other genetic kidney diseases. The establishment of large cohorts of CAKUT will provide sufficient resolution to attribute causality in abnormal kidney and urinary tract development. We believe now is the time to initiate such multi-angled, detailed, and diverse international data- and biobank frameworks to meet the challenge of improving the clinical outcome of CAKUT patients.

## Key summary points

1. The spectrum of CAKUT phenotypes is characterized by an extremely heterogeneous molecular basis and high clinical variability.
2. Despite the incredible advancements in genomic techniques, a genetic diagnosis for CAKUT can currently be established in ~ 5–20% of affected individuals.
3. Environmental factors and their interaction with the genome underlie aberrant kidney and urinary tract development, but their contribution remains largely inconsistent and the molecular mechanisms are unclear.
4. Improvement of translational technologies in the upcoming years will lead to better validation approaches and, as such, improved understanding of the pathomechanisms for CAKUT.
5. Large-scale and world-wide efforts are unequivocally needed to uncover the etiology of CAKUT. Now is the time for our community to initiate this.

## Multiple choice questions

Answers are given following the references.

1. Which is the most realistic proportion for all CAKUT patients with a monogenic diagnosis in current practice?
  - 12%
  - 35%
  - 50%
  - 75%
2. What is meant by variable expressivity in CAKUT?
  - Genetic variants can interact with environmental factors to determine the phenotype
  - Identical disease-causing variants could lead to different phenotypes
  - All CAKUT cases have the same genetic variation
  - CAKUT is caused only by environmental factors
3. Which study characteristics complicate defining the role of common variants in the etiology of CAKUT?
  - High phenotypic heterogeneity
  - Low sample sizes
  - Both a and b
  - None of the above
4. Which environmental factor is shown to be truly causal for CAKUT?
  - Gestational diabetes mellitus
  - Obesity
  - Smoking
  - Causality is not definitively proven for gestational diabetes mellitus, obesity, or smoking
5. In vivo modeling of a candidate genetic variant requires by definition that the specific gene of interest is part of the natural genome of the proposed model system.
  - True
  - False

## Supplementary information

Below is the link to the electronic supplementary material.

Graphical abstract (PPTX 415 KB)

Supplementary file1 (DOCX 66 KB)
