# Supplementary material for: Translational strategies to uncover the etiology of congenital anomalies of the kidney and urinary tract
Source: Pediatr Nephrol. 2024 Oct 7;40(3):685–99. doi: 10.1007/s00467-024-06479-2 (PMC11753331; doi:10.1007/s00467-024-06479-2)
Supplement: Supplementary file 1 — Graphical abstract (PPTX 415 KB) [file 467_2024_6479_MOESM1_ESM.pptx]

## Slide 1
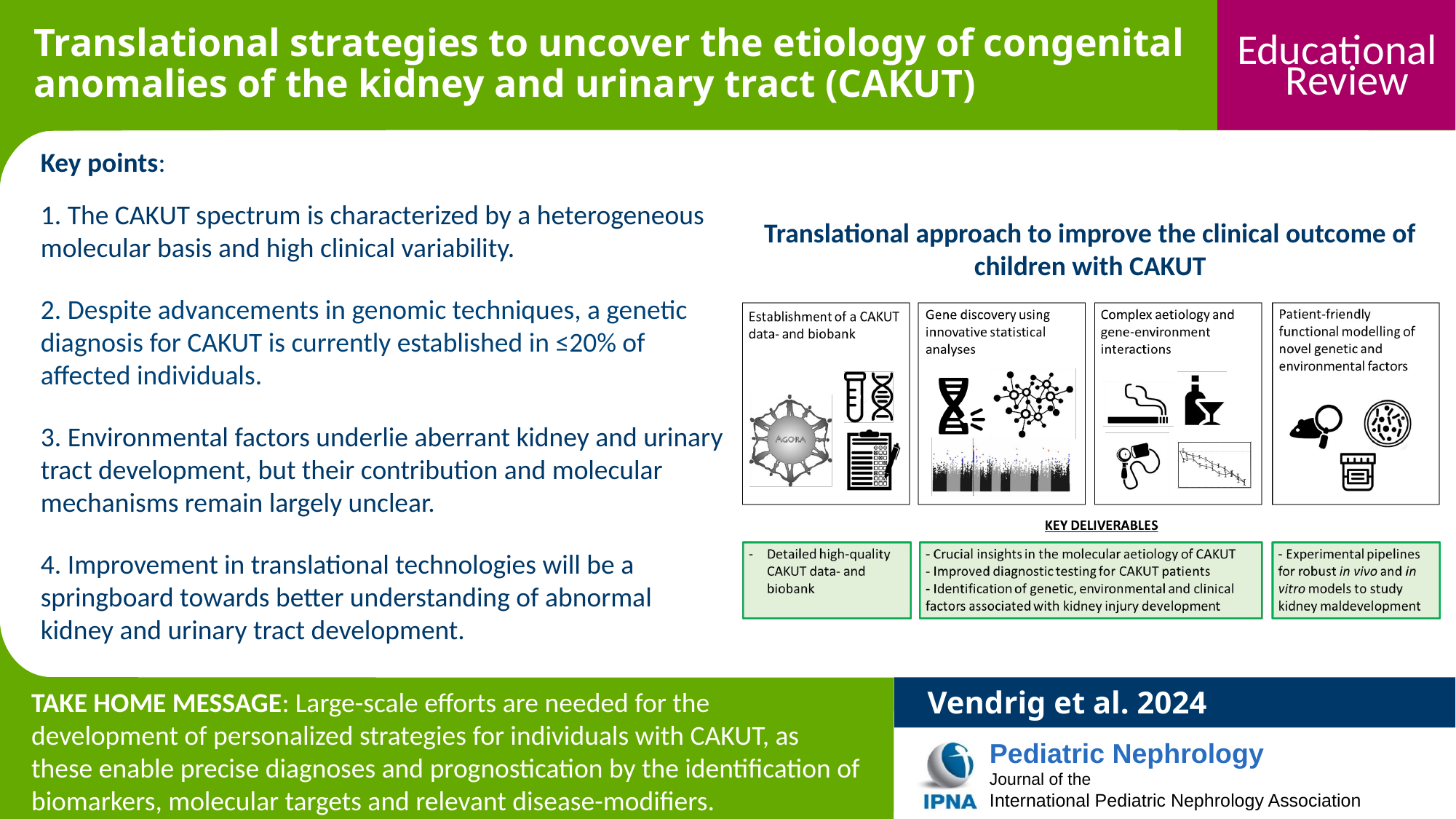

Translational strategies to uncover the etiology of congenital anomalies of the kidney and urinary tract (CAKUT)
Key points:
1. The CAKUT spectrum is characterized by a heterogeneous molecular basis and high clinical variability.
2. Despite advancements in genomic techniques, a genetic diagnosis for CAKUT is currently established in ≤20% of affected individuals.
3. Environmental factors underlie aberrant kidney and urinary tract development, but their contribution and molecular mechanisms remain largely unclear.
4. Improvement in translational technologies will be a springboard towards better understanding of abnormal kidney and urinary tract development.
Translational approach to improve the clinical outcome of children with CAKUT
Vendrig et al. 2024
TAKE HOME MESSAGE: Large-scale efforts are needed for the development of personalized strategies for individuals with CAKUT, as these enable precise diagnoses and prognostication by the identification of biomarkers, molecular targets and relevant disease-modifiers.
