# Supplementary material for: Translational strategies to uncover the etiology of congenital anomalies of the kidney and urinary tract
Source: Pediatr Nephrol. 2024 Oct 7;40(3):685–99. doi: 10.1007/s00467-024-06479-2 (PMC11753331; doi:10.1007/s00467-024-06479-2)
Supplement: Supplementary file 2 — Supplementary file1 (DOCX 66 KB) [file 467_2024_6479_MOESM2_ESM.docx]

**SUPPLEMENTARY TABLE 1. Established genes associated with CAKUT**

| OMIM | Gene symbol | Phenotype | Mode of inheritance |
| --- | --- | --- | --- |
| #106180 | *ACE* | Renal tubular dysgenesis | Autosomal recessive |
| #106150 | *AGT* | Renal tubular dysgenesis | Autosomal recessive |
| #106165 | *AGTR1* | Renal tubular dysgenesis | Autosomal recessive |
| #608669 | *BNC2* | Lower urinary tract obstruction, congenital | Autosomal dominant |
| #300708 | *CCNQ* | STAR syndrome | X-linked dominant |
| #610000 | *CEP55* | Multinucleated neurons, anhydramnios, renal dysplasia, cerebellar hypoplasia, and hydranencephaly | Autosomal recessive |
| #608892 | *CHD7* | CHARGE syndrome | Autosomal dominant |
| #617057 | *CTU2* | Microcephaly, facial dysmorphism, renal agenesis, and ambiguous genitalia syndrome | Autosomal recessive |
| #602858 | *DHCR7* | Smith-Lemli-Opitz syndrome | Autosomal recessive |
| #612666 | *DSTYK* | CAKUT (hypoplasia, congenital obstructive uropathy) | Autosomal dominant |
| #601653 | *EYA1* | Branchio-oto-renal syndrome 1, with or without cataracts | Autosomal dominant |
| #605558 | *FGF20* | Renal hypodysplasia/aplasia 2 | Autosomal recessive |
| #607830 | *FRAS1* | Fraser syndrome 1 | Autosomal recessive |
| #608944 | *FREM1* | Bifid nose with or without anorectal and renal anomalies | Autosomal recessive |
| #608945 | *FREM2* | Fraser syndrome 2 | Autosomal recessive |
| #131320 | *GATA3* | Hypoparathyroidism, sensorineural deafness, and renal dysplasia | Autosomal dominant |
| #601496 | *GFRA1* | Renal hypodysplasia/aplasia 4 | Autosomal recessive |
| #165240 | *GLI3* | Pallister-Hall syndrome | Autosomal dominant |
| #300037 | *GPC3* | Simpson-Golabi-Behmel syndrome, type 1 | X-linked recessive |
| #617782 | *GREB1L* | Renal hypodysplasia/aplasia 3 | Autosomal dominant |
| #604597 | *GRIP1* | Fraser syndrome 3 | Autosomal recessive |
| #604521 | *HAAO* | Vertebral, cardiac, renal, and limb defects syndrome 1 | Autosomal recessive |
| #189907 | *HNF1B* | Renal cysts and diabetes syndrome | Autosomal dominant |
| #142959 | *HOXA13* | Hand-foot-genital syndrome | Autosomal dominant |
| #613469 | *HPSE2* | Urofacial syndrome 1 | Autosomal recessive |
| #604063 | *ITGA8* | Renal hypodysplasia/aplasia 1 | Autosomal recessive |
| #601920 | *JAG1* | Alagille syndrome 1 | Autosomal dominant |
| #300128 | *KDM6A* | Kabuki syndrome 2 | X-linked dominant |
| #602113 | *KMT2D* | Kabuki syndrome 1 | Autosomal dominant |
| #605197 | *KYNU* | Vertebral, cardiac, renal, and limb defects syndrome 2 | Autosomal recessive |
| #608869 | *LRIG2* | Urofacial syndrome 2 | Autosomal recessive |
| #604270 | *LRP4* | Cenani-Lenz syndactyly syndrome | Autosomal recessive |
| #606127 | *MYOCD* | Megabladder, congenital | Autosomal dominant |
| #608285 | *NADSYN1* | Vertebral, cardiac, renal, and limb defects syndrome 3 | Autosomal recessive |
| #608667 | *NIPBL* | Cornelia de Lange syndrome 1 | Autosomal dominant |
| #600275 | *NOTCH2* | Alagille syndrome 2 | Autosomal dominant |
| #602490 | *NRIP1* | CAKUT (hypodysplasia, vesicoureteral reflux, megaureters, hydronephrosis, multicystic dysplastic kidneys, and small kidneys, kidney ectopia) | Autosomal dominant |
| #167409 | *PAX2* | Papillorenal syndrome | Autosomal dominant |
| #176310 | *PBX1* | Congenital anomalies of kidney and urinary tract syndrome with or without hearing loss, abnormal ears, or developmental delay | Autosomal dominant |
| #607647 | *PLVAP* | Diarrhea 10, protein-losing enteropathy type | Autosomal recessive |
| #179820 | *REN* | Renal tubular dysgenesis | Autosomal recessive |
| #164761 | *RET* | Multiple endocrine neoplasia IIA | Autosomal dominant |
| #602430 | *ROBO1* | Neurooculorenal syndrome | Autosomal recessive |
| #602431 | *ROBO2* | Vesicoureteral reflux 2 | Autosomal dominant |
| #602337 | *ROR2* | Robinow syndrome, autosomal recessive | Autosomal recessive |
| #602218 | *SALL1* | Townes-Brocks branchiootorenal-like syndrome | Autosomal dominant |
| #601205 | *SIX1* | Branchiootic syndrome 3 | Autosomal dominant |
| #600963 | *SIX5* | Branchiootorenal syndrome 2 | Autosomal recessive |
| #610928 | *SOX17* | Vesicoureteral reflux 3 | Autosomal dominant |
| #610745 | *STRA6* | Microphthalmia, isolated, with coloboma 8 | Autosomal recessive |
| #604613 | *TBX18* | CAKUT (ureteropelvic junction obstruction, pelviureteric junction obstruction, hydronephrosis due to pelviureteric junction obstruction, bilateral multicystic dysplastic kidneys) | Autosomal dominant |
| #107580 | *TFAP2A* | Branchio-oculo-facial syndrome | Autosomal dominant |
| #617449 | *TMEM260* | Structural heart defects and renal anomalies syndrome | Autosomal recessive |
| #600985 | *TNXB* | Vesicoureteral reflux 8 | Autosomal dominant |
| #606219 | *TRAP1* | VACTERL association | Autosomal recessive |
| #618083 | *WBP11* | Vertebral, cardiac, tracheoesophageal, renal, and limb defects | Autosomal dominant |
| #603490 | *WNT4* | Mullerian aplasia and hyperandrogenism | Autosomal dominant |
| #300265 | *ZIC3* | VACTERL association, X-linked | X-linked recessive |
| #602221 | *ZMYM2* | Neurodevelopmental-craniofacial syndrome with variable renal and cardiac abnormalities | Autosomal dominant |
| Genes that may be associated with CAKUT, but convincing genotype-phenotype correlation is lacking | | | |
| #102545 | *ACTG2* | Megacystis-microcolon-intestinal hypoperistalsis syndrome 5 | Autosomal dominant |
| #118494 | *CHRM3* | Prune belly syndrome | Autosomal recessive |
| #601090 | *FOXC1* | Axenfeld-Rieger syndrome, type 3 and Anterior segment dysgenesis 3, multiple subtypes | Autosomal dominant |
| #151443 | *LIFR* | Stuve-Wiedemann syndrome/Schwartz-Jampel type 2 syndrome | Autosomal recessive |
| #608002 | *NPHP3* | Meckel syndrome 7, Nephronophthisis 3 and Renal-hepatic-pancreatic dysplasia 1 | Autosomal recessive |
| #610306 | *NPNT* | Bilateral kidney agenesis | Autosomal recessive |
| #604994 | *SIX2* | NA | NA |
| #603746 | *SLIT2* | CAKUT (cystic dysplastic kidneys, unilateral kidney agenesis, duplicated collecting system) | Autosomal dominant |
| #606523 | *SRGAP1* | Thyroid cancer, nonmedullary, 2 | Autosomal dominant |
| #609850 | *TBC1D1* | CAKUT (bilateral kidney  (hypo)dysplasia combined with urinary tract dilation, unilateral kidney hypodysplasia combined with unilateral renal agenesis, Mayer–Rokitansky–Küster–Hauser syndrome | Autosomal dominant |
| #611559 | *UPK3A* | Vesicoureteral reflux, duplex collecting system | Autosomal dominant |
| Examples of genes where the correlation with CAKUT is described [13] | | | |
| #602211 | *FOXD2* | Syndromic CAKUT (uni-/bilateral hypoplastic kidneys, bilateral dysplastic kidneys) | Autosomal recessive |
| #604264 | *CELSR3* | NA | NA |

This Table contains genes that are listed in two or more of the following sources: 1) genes listed as green in the CAKUT panel (version 1.177) from Genomics England PanelApp, 2) the diagnostic CAKUT gene panel from UMC Utrecht, version NEF03v23.2, and, 3) Table S1 (54 genes representing monogenic causes of isolated or syndromic human CAKUT, if mutated) from Kolvenbach et al. [13]. It is important to note that we aimed because of the scope of this Review to include only genes correlated with primary CAKUT, and removed phenocopies (like ciliopathies). However, phenocopies are highly relevant for diagnostic gene panel design. In case phenotype and/or mode of inheritance were unavailable at these sources, additional studies were analyzed by using PubMedd and EMBASE. NA = not available; OMIM = Online Mendelian Inheritance in Man.

| **SUPPLEMENTARY TABLE 2.** Curated list of genomic disorders associated with CAKUT[14] | | | | |  |  |  |  |  |  |
| --- | --- | --- | --- | --- | --- | --- | --- | --- | --- | --- |
|  |  |  |  |  |  |  |  |  |  |  |
| **Chromosome** | **Start (Mb)** | **End (Mb)** | **Size (Mb)** | **Syndrome name** |  |  |  |  |  |  |
| 1 | 89.7 | 90.2 | 0.5 | 1p22 duplication |  |  |  |  |  |  |
| 1 | 0.0 | 12.8 | 12.8 | 1p36 duplication |  |  |  |  |  |  |
| 1 | 2.9 | 3.7 | 0.7 | 1p36 duplication |  |  |  |  |  |  |
| 1 | 0.0 | 12.8 | 12.8 | 1p36 microdeletion syndrome |  |  |  |  |  |  |
| 1 | 146.5 | 147.9 | 1.3 | 1q21.1 recurrent microdeletion |  |  |  |  |  |  |
| 1 | 146.5 | 147.9 | 1.3 | 1q21.1 recurrent microduplication |  |  |  |  |  |  |
| 1 | 145.4 | 145.7 | 0.4 | 1q21.1 susceptibility locus for the thrombocytopenia-absent radius (TAR) syndrome |  |  |  |  |  |  |
| 1 | 145.4 | 145.7 | 0.4 | 1q21.1 thromobcytopenia-absent radius syndrome (TAR) region duplication |  |  |  |  |  |  |
| 1 | 242.5 | 247.6 | 5.1 | 1q43-q44 deletion |  |  |  |  |  |  |
| 2 | 59.3 | 61.8 | 2.5 | 2p15-p16.1 microdeletion syndrome |  |  |  |  |  |  |
| 2 | 44.4 | 44.6 | 0.2 | 2p21 microdeletion syndrome |  |  |  |  |  |  |
| 2 | 96.7 | 97.7 | 0.9 | 2q11.2 deletion |  |  |  |  |  |  |
| 2 | 96.7 | 97.7 | 0.9 | 2q11.2 duplication |  |  |  |  |  |  |
| 2 | 100.7 | 108.4 | 7.8 | 2q11.2-q13 deletion |  |  |  |  |  |  |
| 2 | 100.7 | 108.4 | 7.8 | 2q11.2-q13 duplication |  |  |  |  |  |  |
| 2 | 110.8 | 111.0 | 0.2 | 2q13 homozygous deletion nephronophtisis 1 |  |  |  |  |  |  |
| 2 | 145.1 | 145.3 | 0.1 | 2q22 Mowat-Wilson syndrome |  |  |  |  |  |  |
| 2 | 148.7 | 149.3 | 0.6 | 2q23 duplication |  |  |  |  |  |  |
| 2 | 148.7 | 149.3 | 0.6 | 2q23.1 deletion |  |  |  |  |  |  |
| 2 | 196.9 | 205.2 | 8.3 | 2q33.1 deletion syndrome |  |  |  |  |  |  |
| 2 | 239.7 | 242.5 | 2.8 | 2q37 deletion |  |  |  |  |  |  |
| 2 | 241.3 | 242.8 | 1.5 | 2q37 deletion |  |  |  |  |  |  |
| 2 | 240.0 | 240.3 | 0.4 | 2q37 monosomy |  |  |  |  |  |  |
| 3 | 1.4 | 2.2 | 0.8 | 3pterm-p25 deletion |  |  |  |  |  |  |
| 3 | 195.7 | 197.3 | 1.6 | 3q29 microdeletion syndrome |  |  |  |  |  |  |
| 3 | 195.7 | 197.3 | 1.6 | 3q29 microduplication syndrome |  |  |  |  |  |  |
| 3 | 147.0 | 147.2 | 0.2 | Dandy-Walker syndrome critical region deletion |  |  |  |  |  |  |
| 3 | 147.0 | 147.2 | 0.2 | Dandy-Walker syndrome critical region duplication |  |  |  |  |  |  |
| 4 | 111.4 | 111.6 | 0.2 | Axenfeld-Rieger syndrome deletion |  |  |  |  |  |  |
| 4 | 0.1 | 17.7 | 17.6 | Wolf-Hirschhorn deletion syndrome |  |  |  |  |  |  |
| 4 | 1.9 | 2.0 | 0.1 | Wolf-Hirschhorn critical deletion |  |  |  |  |  |  |
| 5 | 1.9 | 2.0 | 0.1 | Familial Adenomatous Polyposis duplication |  |  |  |  |  |  |
| 5 | 0.1 | 10.9 | 10.8 | 5p distal duplication |  |  |  |  |  |  |
| 5 | 36.9 | 37.1 | 0.2 | 5p13.2 Cornelia de Lange syndrome |  |  |  |  |  |  |
| 5 | 91.4 | 114.5 | 23.1 | 5q interstitial deletion |  |  |  |  |  |  |
| 5 | 126.1 | 126.2 | 0.1 | Adult-onset autosomal dominant leukodystrophy duplication |  |  |  |  |  |  |
| 5 | 0.0 | 12.5 | 12.5 | Cri du Chat Syndrome (5p deletion) |  |  |  |  |  |  |
| 5 | 112.0 | 112.2 | 0.1 | Familial Adenomatous Polyposis deletion |  |  |  |  |  |  |
| 5 | 175.7 | 177.1 | 1.3 | Sotos syndrome deletion |  |  |  |  |  |  |
| 6 | 45.3 | 45.5 | 0.2 | 6p21.1 Cleidocranial dysplasia (CCD) |  |  |  |  |  |  |
| 6 | 70.2 | 70.7 | 0.5 | 6q13-q14 deletion |  |  |  |  |  |  |
| 7 | 6.9 | 7.3 | 0.4 | 7p interstitial duplication |  |  |  |  |  |  |
| 7 | 42.0 | 42.3 | 0.3 | 7p14.1 Greig cephalopolysyndactyly |  |  |  |  |  |  |
| 7 | 23.7 | 27.5 | 3.7 | 7p15 deletion |  |  |  |  |  |  |
| 7 | 16.8 | 17.7 | 0.9 | 7p21 interstitial duplication |  |  |  |  |  |  |
| 7 | 72.7 | 74.1 | 1.4 | 7q11.23 duplication syndrome |  |  |  |  |  |  |
| 7 | 152.6 | 159.1 | 6.5 | 7q36 deletion critical |  |  |  |  |  |  |
| 7 | 141.9 | 159.1 | 17.2 | 7q36.1 deletion |  |  |  |  |  |  |
| 7 | 75.0 | 76.7 | 1.7 | Williams-Beuren syndrome distal deletion |  |  |  |  |  |  |
| 7 | 75.0 | 76.7 | 1.7 | Williams-Beuren syndrome distal duplication |  |  |  |  |  |  |
| 7 | 72.7 | 74.1 | 1.4 | Williams-Beuren syndrome |  |  |  |  |  |  |
| 8 | 8.1 | 11.9 | 3.8 | 8p23.1 deletion |  |  |  |  |  |  |
| 8 | 8.1 | 11.9 | 3.8 | 8p23.1 duplication |  |  |  |  |  |  |
| 8 | 72.1 | 72.3 | 0.2 | 8q13.3 branchio-oto-renal/Melnick-Fraser/oto-facio-cervical syndrome |  |  |  |  |  |  |
| 8 | 77.2 | 77.8 | 0.5 | 8q21.11 microdeletion syndrome |  |  |  |  |  |  |
| 9 | 14.8 | 15.0 | 0.2 | 9p22 deletion |  |  |  |  |  |  |
| 9 | 140.5 | 140.7 | 0.2 | 9q subtelomeric deletion syndrome |  |  |  |  |  |  |
| 9 | 98.2 | 98.3 | 0.1 | 9q22.32 basal cell nevus/Gorlin-Goltz/holoprosencephaly |  |  |  |  |  |  |
| 9 | 129.4 | 129.5 | 0.1 | 9q33.3 nail-patella syndrome |  |  |  |  |  |  |
| 9 | 137.8 | 141.1 | 3.3 | 9q34 deletion |  |  |  |  |  |  |
| 9 | 137.8 | 141.1 | 3.3 | 9q34 duplication |  |  |  |  |  |  |
| 10 | 82.0 | 88.8 | 6.8 | 10q23 deletion |  |  |  |  |  |  |
| 10 | 82.0 | 88.8 | 6.8 | 10q23 duplication |  |  |  |  |  |  |
| 10 | 9.8 | 12.2 | 2.3 | DiGeorge syndrome locus 2 deletion |  |  |  |  |  |  |
| 10 | 9.8 | 12.2 | 2.3 | DiGeorge syndrome locus 2 duplication |  |  |  |  |  |  |
| 10 | 8.0 | 8.2 | 0.2 | hypoparathyroidism. sensorineural deafness. and renal disease deletion |  |  |  |  |  |  |
| 10 | 8.0 | 8.2 | 0.2 | hypoparathyroidism. sensorineural deafness. and renal disease duplication |  |  |  |  |  |  |
| 11 | 44.0 | 46.1 | 2.1 | 11p11.2 duplication |  |  |  |  |  |  |
| 11 | 2.0 | 2.8 | 0.9 | Beckwith-Wiedeman syndrome deletion |  |  |  |  |  |  |
| 11 | 2.0 | 2.8 | 0.9 | Beckwith-Wiedeman syndrome duplication |  |  |  |  |  |  |
| 11 | 128.5 | 134.7 | 6.1 | Jacobsen syndrome deletion |  |  |  |  |  |  |
| 11 | 128.5 | 134.7 | 6.1 | Jacobsen syndrome duplication |  |  |  |  |  |  |
| 11 | 44.0 | 46.1 | 2.1 | Potocki-Schaffer syndrome deletion |  |  |  |  |  |  |
| 11 | 67.8 | 71.3 | 3.5 | SHANK FGFs deletion |  |  |  |  |  |  |
| 11 | 31.8 | 32.5 | 0.7 | WAGR 11p13 deletion syndrome |  |  |  |  |  |  |
| 12 | 65.1 | 68.6 | 3.6 | 12q14 microdeletion syndrome |  |  |  |  |  |  |
| 15 | 22.7 | 28.4 | 5.7 | 15q11.2 Prader-Willi/Angelman (Type 1) deletion |  |  |  |  |  |  |
| 15 | 22.7 | 28.4 | 5.7 | 15q11.2 Prader-Willi/Angelman (Type 1) reciprocal duplication |  |  |  |  |  |  |
| 15 | 23.6 | 28.4 | 4.8 | 15q11.2 Prader-Willi/Angelman syndrome (Type 2) deletion |  |  |  |  |  |  |
| 15 | 23.6 | 28.4 | 4.8 | 15q11.2 Prader-Willi/Angelman region reciprocal duplication |  |  |  |  |  |  |
| 15 | 31.1 | 32.5 | 1.4 | 15q13.3 duplication |  |  |  |  |  |  |
| 15 | 30.9 | 32.4 | 1.5 | 15q13.3 microdeletion syndrome |  |  |  |  |  |  |
| 15 | 72.9 | 74.4 | 1.5 | 15q24 BP0-BP1 deletion |  |  |  |  |  |  |
| 15 | 72.9 | 74.4 | 1.5 | 15q24 BP0-BP1 duplication |  |  |  |  |  |  |
| 15 | 72.9 | 75.8 | 2.9 | 15q24 BP0-BP1b deletion |  |  |  |  |  |  |
| 15 | 72.9 | 75.8 | 2.9 | 15q24 BP0-BP1b duplication |  |  |  |  |  |  |
| 15 | 76.0 | 78.2 | 2.2 | 15q24 BP2-BP3 deletion |  |  |  |  |  |  |
| 15 | 83.1 | 84.7 | 1.6 | 15q25.2 deletion |  |  |  |  |  |  |
| 15 | 99.4 | 102.5 | 3.2 | 15q26 overgrowth syndrome deletion |  |  |  |  |  |  |
| 15 | 99.4 | 102.5 | 3.2 | 15q26 overgrowth syndrome duplication |  |  |  |  |  |  |
| 16 | 0.1 | 15.2 | 15.1 | 16p subtelomeric duplication |  |  |  |  |  |  |
| 16 | 29.7 | 30.2 | 0.6 | 16p11.2 microdeletion |  |  |  |  |  |  |
| 16 | 29.7 | 30.2 | 0.6 | 16p11.2 duplication |  |  |  |  |  |  |
| 16 | 21.4 | 29.4 | 8.1 | 16p11.2-p12.1 deletion |  |  |  |  |  |  |
| 16 | 21.4 | 29.4 | 8.1 | 16p11.2-p12.1 duplication |  |  |  |  |  |  |
| 16 | 15.1 | 15.9 | 0.8 | 16p13.11 duplication |  |  |  |  |  |  |
| 16 | 15.1 | 15.9 | 0.8 | 16p13.11 duplication |  |  |  |  |  |  |
| 16 | 15.5 | 16.3 | 0.8 | 16p13.11 recurrent microdeletion (neurocognitive disorder susceptibility locus) |  |  |  |  |  |  |
| 16 | 15.5 | 16.3 | 0.8 | 16p13.11 recurrent microduplication (neurocognitive disorder susceptibility locus) |  |  |  |  |  |  |
| 16 | 0.1 | 0.8 | 0.8 | ATR-16 syndrome |  |  |  |  |  |  |
| 16 | 3.8 | 3.9 | 0.2 | Rubinstein-Taybi Syndrome |  |  |  |  |  |  |
| 17 | 0.1 | 2.6 | 2.5 | 17p13.3 duplication YWHAE and PAFAH1B1 |  |  |  |  |  |  |
| 17 | 34.8 | 36.2 | 1.4 | 17q12 duplication |  |  |  |  |  |  |
| 17 | 43.7 | 44.3 | 0.6 | 17q21.31 duplication |  |  |  |  |  |  |
| 17 | 43.7 | 44.3 | 0.6 | 17q21.31 recurrent microdeletion syndrome |  |  |  |  |  |  |
| 17 | 57.7 | 58.1 | 0.4 | 17q23 deletion type 1 |  |  |  |  |  |  |
| 17 | 58.1 | 60.3 | 2.2 | 17q23 deletion type 2 |  |  |  |  |  |  |
| 17 | 14.1 | 15.5 | 1.4 | Charcot-Marie-Tooth syndrome type 1A (CMT1A) |  |  |  |  |  |  |
| 17 | 3.5 | 3.6 | 0.1 | Cystinosin deletion |  |  |  |  |  |  |
| 17 | 14.1 | 15.5 | 1.4 | Hereditary liability to pressure palsies (HNPP) |  |  |  |  |  |  |
| 17 | 0.0 | 2.6 | 2.6 | Miller-Dieker syndrome (MDS) |  |  |  |  |  |  |
| 17 | 29.2 | 30.2 | 1.1 | Neurofibromatosis 1 duplication |  |  |  |  |  |  |
| 17 | 29.2 | 30.2 | 1.1 | Neurofibromatosis 1-microdeletion syndrome |  |  |  |  |  |  |
| 17 | 16.7 | 20.5 | 3.8 | Potocki-Lupski syndrome duplication |  |  |  |  |  |  |
| 17 | 34.8 | 36.2 | 1.4 | Renal cysts and diabeters deletion |  |  |  |  |  |  |
| 17 | 16.7 | 20.5 | 3.8 | Smith-Magenis syndrome deletion |  |  |  |  |  |  |
| 20 | 0.2 | 24.8 | 24.7 | 20p partial trisomy |  |  |  |  |  |  |
| 21 | 41.6 | 48.1 | 6.5 | 21q partial monosomy |  |  |  |  |  |  |
| 21 | 27.3 | 27.5 | 0.3 | Early-onset Alzheimer disease with cerebral amyloid angiopathy |  |  |  |  |  |  |
| 22 | 21.9 | 23.7 | 1.7 | 22q11.2 distal duplication |  |  |  |  |  |  |
| 22 | 18.9 | 21.5 | 2.6 | 22q11.21 duplication (velo-cardial-facial syndrome region) |  |  |  |  |  |  |
| 22 | 30.0 | 30.1 | 0.1 | 22q12.2 Neurofibromatosis 2 |  |  |  |  |  |  |
| 22 | 51.0 | 51.2 | 0.1 | 22q13.3-q13.3 Microdeletion |  |  |  |  |  |  |
| 22 | 18.9 | 20.3 | 1.4 | DiGeorge (LCR A-B) |  |  |  |  |  |  |
| 22 | 18.9 | 21.5 | 2.6 | DiGeorge (LCR A-D) |  |  |  |  |  |  |
| 22 | 20.7 | 21.5 | 0.7 | DiGeorge (LCR B-D) |  |  |  |  |  |  |
| 22 | 21.1 | 21.5 | 0.4 | DiGeorge (LCR C-D) |  |  |  |  |  |  |
| 22 | 21.1 | 22.9 | 1.8 | DiGeorge (LCR C-E) |  |  |  |  |  |  |
| 22 | 21.9 | 22.9 | 1.0 | DiGeorge (LCR D-E) |  |  |  |  |  |  |
| 22 | 21.9 | 23.6 | 1.7 | DiGeorge (LCR D-F) |  |  |  |  |  |  |
| 22 | 21.9 | 25.0 | 3.1 | DiGeorge (LCR D-H) |  |  |  |  |  |  |
| 22 | 22.9 | 25.0 | 2.1 | DiGeorge (LCR E-H) |  |  |  |  |  |  |
| 22 | 23.7 | 25.0 | 1.3 | DiGeorge (LCR F-H) |  |  |  |  |  |  |
| 22 | 44.6 | 51.2 | 6.6 | Phelan-McDermid syndrome |  |  |  |  |  |  |
| X | 103.7 | 110.6 | 6.9 | Alport syndrome-mental-retardation-midface hypoplasia-elliptocytosis syndrome |  |  |  |  |  |  |
| X | 0.8 | 0.9 | 0.1 | Leri-Weill dyschondrostosis - SHOX deletion type 1 |  |  |  |  |  |  |
| X | 0.5 | 0.8 | 0.3 | Leri-Weill dyschondrostosis - SHOX deletion type 2 |  |  |  |  |  |  |
| X | 139.5 | 140.1 | 0.5 | Mental retardation with panhypopituitarism syndrome duplication |  |  |  |  |  |  |
| X | 6.5 | 8.1 | 1.7 | Steroid sulphatase deficiency deletion |  |  |  |  |  |  |
| X | 0.1 | 155.3 | 155.2 | Triple X syndrome |  |  |  |  |  |  |
| X | 53.4 | 53.7 | 0.3 | Xp11.22-linked intellectual disability duplication |  |  |  |  |  |  |
| X | 31.1 | 33.4 | 2.2 | Xp21.2 Duchenne region |  |  |  |  |  |  |
| X | 30.7 | 30.7 | 0.1 | Xp21.2 Glycerol kinase deficiency |  |  |  |  |  |  |
| X | 8.5 | 8.7 | 0.2 | Xp22.31 Kallmann syndrome deletion |  |  |  |  |  |  |
| X | 110.5 | 110.7 | 0.1 | Xq23 X-linked lissencephaly |  |  |  |  |  |  |
| X | 153.3 | 153.4 | 0.1 | Xq28 duplication |  |  |  |  |  |  |
| X | 153.6 | 153.9 | 0.3 | Xq28 microduplication |  |  |  |  |  |  |
| X | 153.3 | 153.4 | 0.1 | Xq28 Rett syndrome deletion |  |  |  |  |  |  |
| Coordinates are based on hg19. LCR = low-copy repeat. | | | | |  |  |  |  |  |  |

**SUPPLEMENTARY DATA. Genetic definitions in the context of CAKUT etiology**

- **Syndromal CAKUT** is defined by one of more CAKUT manifestations in combination with extra-renal anomalies.
- **Whole Exome Sequencing (WES)** is a method of sequencing exons or protein-coding regions of a genome.
- **Whole genome sequencing (WGS)** is a method of sequencing the entire genetic make-up of an organism, including both coding and non-coding regions of the DNA.
- **Single nucleotide variant (SNV)** is a variant in which a single nucleotide (A, T, C or G) in the DNA sequence is altered.
- **Copy number variant (CNV)** is an abnormal number of copies of one or more sections of DNA.
- **Structural variant (SV)** is a large variant in the genome that can include deletions and duplications. but also inversions and translocations of DNA segments.
- **Epigenetic variation** encompasses differences in gene expression patterns among cells or organisms that are caused by epigenetic modifications (such as DNA methylation or histone modification) rather than changes in the DNA sequence.
- **Genome-Wide Association Study (GWAS)** identifies associations between common genetic markers, typically single nucleotide polymorphisms (SNPs) and particular diseases or traits.
- **Minor allele frequency (MAF)** is the frequency at which the less common variant (allele) of a genetic marker occurs in a given population.
- **Gene-environment interaction** describes how the impact of an individual's genetic makeup on their traits or disease risk is modified by environmental factors.
- ***In silico*** predictions refer to computational simulations and models used to predict biological outcomes based on virtual experiments and data analysis.
- **Functional validation** is the process of experimentally confirming the biological activity or role of a gene, protein, or genetic variant in a specific cellular or organismal context.
- ***In vitro*** cell culture is the technique of growing and maintaining cells in a controlled artificial environment outside their natural biological context.
- **Organoids** are three-dimensional and simplified versions of organs produced *in vitro* from stem cells, which mimic the structure and function of real organs.
- The **transcriptome** is the complete set of RNA transcripts produced by the genome.
- The **proteome** is the entire set of proteins expressed by a genome, cell, tissue or organism.
